# The Socio-Cultural Factors Influencing the Level of Public Compliance with Infection Control Measures during the 1918 Influenza Pandemic in Italy: A Historical Approach

**DOI:** 10.3390/healthcare12060694

**Published:** 2024-03-20

**Authors:** Eugenia Tognotti, Marco Dettori

**Affiliations:** 1 Center for Anthropological, Paleopathological and Historical Studies of Sardinian and the Mediterranean Populations, Department of Biomedical Sciences, University of Sassari, 07100 Sassari, Italy; tognotti@uniss.it; 2 Department of Medicine, Surgery and Pharmacy, University of Sassari, 07100 Sassari, Italy

**Keywords:** NPIs, 1918 influenza pandemic, Italy, compliance, socio-cultural factors

## Abstract

During health emergencies, non-pharmaceutical interventions (NPIs) are adopted in various combinations until a vaccine can be produced and widely administered. Containment strategies, including the closure of schools, churches, and dance halls; banning of mass gatherings; mandatory mask wearing; isolation; and disinfection/hygiene measures, require reasonable compliance to be successfully implemented. But what are the most effective measures? To date, few systematic studies have been conducted on the effects of various interventions used in past epidemics/pandemics. Important contributions to our understanding of these questions can be obtained by investigating the historical data from the great influenza pandemic of 1918, an event widely considered one of the greatest natural disasters in human history. Taking on particular importance is the study of the possible role played by the behaviour of the population and the lack of public obedience to the non-pharmaceutical interventions in a Mediterranean country like Italy—one of the most affected countries in Europe—during that pandemic, with special attention paid to the weight of the socio-cultural factors which hindered the ultimate goal of containing the spread of the virus and preventing excess deaths in the country.

## 1. Introduction

Non-pharmaceutical interventions (NPIs) are mitigation strategies which have been employed since the late Middle Ages and the Modern Age to control the spread of communicable diseases, epidemics, and pandemics [1]. They include both compulsory measures endorsed by public health regulations, such as the closure of schools and establishments, quarantine/isolation, curfews, severe gathering bans, and restrictions on people’s mobility, and recommended measures, including hand washing and disinfecting surfaces, wearing facial masks, working from home, and keeping a social distance from others.

Some of these measures are thousands of years old. According to ancient historians, one of the first public health measures dates back to the Byzantine emperor Justinian. During a devastating plague epidemic (541–542 A.D.), he imposed isolation measures on travellers and foodstuffs arriving in Constantinople from North Africa to reduce contact in the community, thereby curbing the spread of infection. Responses to public health emergencies have evolved and adapted throughout history [2]. However, it is the 14th-century bubonic plague that set a precedent for the development of a coherent model that would be perfected over the following centuries. Given the lack of medical efficacy at the time, the only way to keep the plague under control and limit its spread was a complex system of quarantines, land and sea sanitary cordons, the isolation of those infected, and fumigation/disinfection [2].

Anybody entering a city was required to present a ‘health card’ issued by the authorities in their place of origin, not dissimilar to the EU digital green certificate (Green Pass) introduced during the COVID-19 pandemic. Non-pharmaceutical interventions, in various combinations and with different adaptations, were also implemented during epidemic waves of yellow fever in the United States and Livorno in 1804 during an epidemic that had been brought from Cadiz by ship. Fearing this exotic disease, the public closely obeyed rules that restricted freedoms, according to the written record of the doctor responsible for managing the crisis. This physician achieved positive results through a combination of quarantining families under control, sanitisation of homes previously occupied by those infected, and the segregation of sick people [3].

Several measures were taken during the various epidemic waves of cholera that swept through Italy from 1835 to the end of the 19th century, as well as during the ‘Russian Flu’ pandemic in 1889–1892 [4]. Twenty years later, they resurfaced with the Spanish flu outbreak in 1918. Due to the lack of effective drugs and vaccines at that time, NPIs played a significant early role in controlling the spread of the disease. It was then that the first masks—gauze cloths covering the nose and mouth—appeared, especially in certain countries. Among the interventions adopted by almost all nations were ‘social distancing’ measures (as we call them today) ranging from closing schools and banning public gatherings to isolating the sick in hospitals or recommending that they stay home from the onset of symptoms [5].

NPIs designed to reduce infectious person-to-person contact have therefore always been an integral part of plans to mitigate the impact of an epidemic/pandemic [6]. The potential benefits of some of these interventions are supported to this day by mathematical models. However, there is a lack of historical evidence of their impact in past pandemics, including the so-called Spanish flu in 1918, an event widely regarded as one of the greatest natural disasters in human history. Attempts at a systematic examination, based on trends in mortality rates, have been made for a variety of associated interventions, such as school, church, and theatre closures and public gathering bans, implemented in a group of 17 US cities [7]. The findings of these and other studies seem to show that a critical factor in the reduction of mortality rates was the speed with which the measures were implemented by the municipalities of several cities (such as St. Louis and Philadelphia) that introduced them following the earliest cases [8].

Such studies do not exist for Italy, one of the worst-affected countries in Europe [9], where the excess mortality rate for the 1918–1919 influenza pandemic was estimated to be 1.1% [10]. The highest excess mortality rate (per 10,000 inhabitants) cumulated throughout the entire excess mortality period was observed in Portugal (233/10,000 inhabitants), followed by Italy (Table 1).

Unlike in other countries [11], the second deadly wave of the disease arrived in Italy in the early autumn, with an often fatal course, and was followed by another, less severe, wave early in 1919 (Table 2).

There was no simultaneous outbreak of Spanish flu throughout the country. The virus first arrived in a few Italian regions in the north and south, and caught the country in an epidemic grip. First, it arrived in Calabria, Campania, and Apulia. Then, as the epidemic thinned out in the south, it moved to central Italy (Latium, Abruzzo, and Marche) and the north, affecting Piedmont, Lombardy, and Veneto [12].

**Figure 1:**
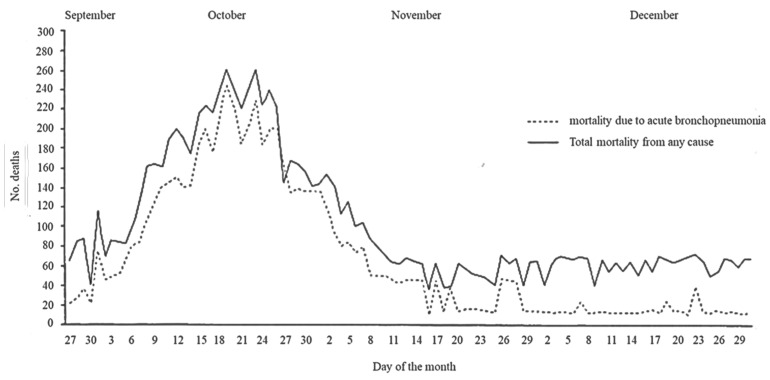
Acute bronchopneumonia mortality and general mortality in Rome between September and December 1918.

The epidemic hit the regions of Italy more or less severely, with higher mortality rates for Lombardy, Lazio, Sardinia, and Basilicata, and lower rates for Veneto, Piedmont, and Liguria. Italy recorded 1.06 deaths per 100 inhabitants, with large regional variations: from a minimum of 0.50 per 100 inhabitants in Veneto to a maximum of 1.19 in Lazio, which included the capital, Rome, one of the hardest-hit cities [12]. An analysis of the rich heritage of historical data, often overlooked, can partially fill the gap left by the lack of targeted studies and offer valuable insights [13] (Figure 1).

The information in the State Archives, Parliamentary Acts, official reports from health authorities, contemporary medical memoirs, medical journals, and newspaper reports all bear unanimous witness to the very low level of public compliance and adherence to the NPIs introduced by the authorities [14]. This is particularly true for the recommendations concerning ‘social distancing’ (as we call it today), which entailed a traumatic change in customs, habits, and rituals (exchanges of greetings and handshakes, visits to the sick, religious ceremonies). Some studies currently in progress will be able to verify the role of traditions and specific socio-cultural and psychological factors influencing their application in the context of a Mediterranean country like Italy. This survey of historical data and documents will serve to provide public health officials with useful indications for future possible emergencies involving viral respiratory diseases.

## 2. Influenza in Italy, 1918

The dominant thesis on the origin of influenza in Italy in 1918 was that the disease was bacterial in origin, and doubts about the possible role of an ultrafiltratable virus were just beginning to emerge [15]. Indeed, in the first official announcements in September—at the onset of the second autumn wave—the health authorities were keen to dispel the ‘fanciful’ popular hearsay regarding the nature of the disease by stating that it was without a shadow of a doubt influenza, known in Italy since the Middle Ages. As with malaria, influenza had taken its name in the 14th century, linked to what was believed to be the cause of the disease: the influence of the stars. In later centuries, it had taken on several names (including Grippe). Only in the 18th century, after the pandemic of 1782, did the name influenza come into common use. The bacteriological revolution of the second half of the 19th century, prompted by Pasteur’s discoveries, advanced new hypotheses about the causes of influenza. In 1892, at the time of the ‘Russian flu’ pandemic, Richard Pfeiffer, a collaborator of Robert Koch, had isolated a bacterium, which he named *Haemophilus influenzae*, postulating—incorrectly—that it was the etiological agent of that pandemic. In 1918, viruses were as yet unknown. Doctors, scientists, and public health officials were convinced that ‘Pfeiffer’s bacillus’ was the pathogen responsible.

**Figure 2:**
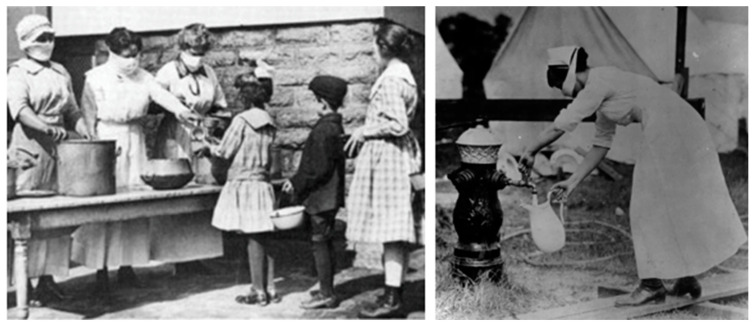
Scenes at the time of the Spanish flu, Italy (source: https://www.fondazionecorriere.corriere.it/ai-tempi-dellinfluenza-spagnola/, accessed on 18 March 2024).

The first public health measures were issued in Italy on 22 August 1918 [16]. In a country at war, measures such as travel restrictions and border controls were unworkable. Thus, quarantine and isolation in ‘lazarettos’, purpose-built in makeshift premises, were only enforced in military training camps where war discipline was in force [17]. It can be assumed that these measures played a part in containing mortality in the combat troops, which was relatively low, according to the observations of contemporaries. Implementing these control measures among the civilian population—which required the sacrifice of individual freedom in the name of a greater good such as health—would have required a very high level of social acceptance and trust in the health authorities (Figure 2).

The implementation of these control measures would also have required the shared expectation that they would be able to control the spread of the infection. The social climate of a war-weary country with little inclination towards other restrictions, however, led the health authorities not to impose these measures. The High Military Command criticised this decision: ‘While the military, in an attempt to stem the progressive weakening of the large Units, resorted to quarantine, albeit with disappointing results, the doctors and civil authorities did not consider setting up either lazarettos, where the infected could be taken, or sanitary cordons to reduce the freedom of movement of citizens’ [18,19]. From the beginning of September, as the pandemic spread, a series of ministerial circulars called on the directors and administrators of communities such as barracks, colleges, schools, and boarding schools to take steps to avoid gatherings and to be vigilant in ensuring strict cleanliness [14]. In the first ten days of October, depending on the local situation, prefects, sub-prefects, and mayors issued decrees ordering the temporary closure of public and private schools, restaurants, and meeting places, and the suspension of public gatherings [20]. In large cities, the prefects—based on Art. 125 of the 1907 Health Law—ordered the closure of theatres and/or a reduction in the number of performances [14,21].

Around mid-October—with the pandemic now at its peak—religious ceremonies and funeral rites were prohibited [16]. In the meantime, the Chief of Public Health Direction distributed its ‘Popular Instructions for Defense against Influenza’, an instruction pamphlet written in simple, easy-to-understand language [22]. These guidelines were prefaced with an instructional note: since the germ of the disease was contained in the mucus of the respiratory tract and was believed to remain there as long as fever and coughing persisted, it was necessary to avoid visiting sick people who shed ‘droplets of saliva’ when coughing or talking. The content and the very order of the recommendations—reported verbatim in Table 3—is instructive, as it indicates the importance attached to individual measures in Italy.

As is apparent from this list—and perhaps drawing on the experience gained in the fight against consumption at the end of the 19th century—reducing contact between the healthy and the sick seems to have been the main concern of the healthcare authorities. Such intention underpinned the instructions ‘not to assemble in enclosed places’ and to maintain a physical distance from patients and convalescents [22]: ‘Healthy people must refrain from visiting or approaching sick or convalescent people unless absolutely necessary’. This went hand in hand with the advice concerning hand, nose, and mouth hygiene and the warning to contain secretions (coughing, sneezing) so as not to spray droplets of saliva [22].

Doctors and nurses were advised to take extra care: hand disinfection with a 1:1000 solution of corrosive sublimate (an inorganic chemical compound of mercury and chlorine) was one of the minimum actions recommended and was also extended to patients’ families [23].

People were also urged to refrain from the widespread habit of spitting on the ground, common among all social classes during the war and post-war years. Mask-wearing is notably absent from the list of precautions. Psychological and cultural factors involving sexual identity also came into play in opposition to masks: for men, hiding their face behind a mask was deemed unmanly, a sign of weakness, and a blow to personal freedom. Against what he called ‘fixations’, Prof. Serafino Belfanti, Director of the Istituto Sieroterapico Milanese (Milanese Serotherapic Institute), lashed out harshly in an article in the Corriere della Sera (Italy’s most authoritative and important newspaper), praising the very different attitude taken by other cultures, such as those of Anglo-Saxon countries. ‘If we Italians,’ he wrote, ‘were to adopt this measure of the handkerchief or the mask, which in my opinion is very effective in defending ourselves from contagion, we would feel ridiculous: in America, hygiene has no such fixations and does not stray from its aims when it believes it is necessary’ [24].

Indeed, only some of the medical staff in hospitals in large cities, according to the documents available, donned ‘special filtering screens to protect themselves from the infected dust’. However, it was only at the height of the epidemic that the Chief of Public Health Direction sent a circular to the prefectures with a sample of ‘anti-flu coverings or masks’ to be used by doctors [14].

Yet further socio-cultural and anthropological factors played a part in the lack of public compliance with the measures adopted by Public Health officials which, despite the constraints imposed by wartime operations, could have slowed the spread of the virus, especially in heavily populated zones. It is worth remembering that hugging and handshaking in greeting was a long-standing ritual of social exchange in Italy. The general non-compliance with the rules of physical distancing recommended by the health authorities was stigmatized by many newspapers, which strongly denounced the custom of shaking hands as ‘a vehicle of evil’ [16,25]. Another recommended measure—that of ‘avoiding the sick, those convalescing from influenza and their belongings’—contrasted with the custom of ‘visiting’ the sick inherited from the charitable ethic of the Christian Middle Ages, which required that relatives and friends visit the homes of those who were bedridden. In fact, the isolation of the sick and convalescent prescribed by the authorities was only possible in hospitals, even though transgressions were frequent despite the strict rules imposed by hospital administrations and the directors of care facilities for the old and infirm [16,21,26].

To overcome these persistent habits, some of the major daily newspapers such as the ‘Nazione’ in Florence and the ‘Corriere della Sera’ endorsed the guidelines issued by the directors of the Municipal Bureau of Hygiene, who recommended avoiding visits to homes and direct contact with flu patients [22]. Adherence to the health authorities’ recommendations found a particular obstacle where religious rituals and rites came into play, such as funeral ceremonies that brought together the deceased’s friends and family in churches and processions to the cemetery, in accordance with a usage particularly rooted in popular southern culture [27].

Dozens and dozens of funerals every day in the cities posed an all-too-dangerous opportunity for contact with possible convalescents and relatives of people who had died of the Spanish flu. In mid-October, at the height of the emergency, the health council of such a major city as Milan issued a recommendation to suspend funeral and burial processions in order to prevent contact, and on the 15th of that month, in agreement with the provincial health council, had ordered the collective transport of corpses to the cemetery. To avoid transgressions, two coffin depositories were set up in Milan, where 127 deaths were recorded on 16 October 1918 alone [28], one at the monumental cemetery and the other at the Porta Romana tram station. No one was permitted to follow the carriage except the priest, who had to remain inside the vehicle. Stopping in the church was not allowed ‘to avoid crowding and so that the religious services (could) take place as an official service even without the presence of the deceased’. The bodies were then taken to the cemetery at night by lorries or by ‘special funeral trams’ [14,29].

These orders had an enormous impact on the collective imagination, inciting mistrust and a collapse in respect for the measures taken by the health authorities: horrific images of dead bodies being carted around the city between 18 and 23 October, sometimes without a casket and wrapped in a simple sheet, aroused indignation and horror. This was echoed in the protests from newspaper readers and in the stances taken by municipal councillors who voiced the public’s opposition to conforming to measures that, although dictated by the higher interest of public health, conflicted with religious beliefs, customs, and habits. A significant observation was made in an appeal to the Mayor asking ‘whether the city administration believed that the forms used to transport corpses (corresponded) to the city’s decorum and the most elementary respect owed to the deceased and their surviving families’ [21,30].

## 3. Conclusions

The introduction of several NPIs in Italy during the 1918 influenza pandemic does not appear to have had a mitigating effect. In terms of the adoption of NPIs and community mitigation strategies, social distancing (which involved the instruction to avoid handshakes and hugs, visits to the sick, and religious services) seems to have been the most disputed by the public. Even in the absence of quantitative data on the effects of non-compliance with the health measures most directly influenced by socio-cultural factors, it can be hypothesised that these played an important role, especially in large cities where the media, albeit conditioned by strict censorship, gave voice to mistrust and protests against certain public health interventions. In preparing for future severe influenza pandemics, these data should also be taken into account when including non-pharmaceutical interventions as complementary measures to the development of effective vaccines and drugs for prophylaxis and treatment. The lessons of 1918, if heeded, could help us avoid repeating the same mistakes in the future, given the real danger in today’s world of many emerging and re-emerging pathogens whose threat is potentially exacerbated by climate change and natural disasters, rapid international travel, mass migration caused by geopolitical upheavals, health inequalities, and public health misinformation [31,32,33].

## Figures and Tables

**Table 1:** Mortality rate attributable to influenza from 1913 to 1920 in the Italian regions [10].

(×10,000 Inhab.)								
	1913	1914	1915	1916	1917	1918	1919	1920
Piedmont	0.7	0.6	0.9	1.3	0.6	64.4	11.2	5.4
Liguria	0.5	0.7	0.7	0.9	1.2	63.8	10.4	7.8
Lombardy	0.8	0.7	0.9	1.7	0.7	72.6	8.6	5.1
Veneto	1.1	0.6	1.3	1.0	0.6	37.9	4.8	4.0
Emilia Romagna	0.9	0.3	0.7	1.2	0.7	67.1	11.0	6.3
Tuscany	0.7	0.4	0.5	0.8	0.5	76.5	9.7	8.4
Marche	1.5	0.8	1.2	1.1	0.8	72.3	12.5	8.8
Umbria	0.9	1.2	1.7	1.3	1.3	73.3	13.8	10.8
Lazio	1.1	0.7	0.4	2.5	0.6	114.7	9.6	8.9
Abruzzo and Molise	2.0	1.4	1.5	1.7	1.5	93.8	8.1	8.6
Campania	1.3	1.0	1.2	1.5	1.2	78.0	8.3	8.5
Apulia	1.7	1.9	1.7	2.8	1.8	89.8	4.8	5.2
Basilicata	3.1	2.5	2.5	4.3	3.0	105.1	16.5	8.6
Calabria	2.4	1.4	1.8	2.6	2.0	104.8	10.4	7.5
Sicily	1.0	1.0	1.0	1.7	0.9	76.1	4.9	4.5
Sardinia	2.7	2.4	2.1	2.1	2.8	108.8	10.7	12.4

**Table 2:** Excess of the number of deaths in 1918 over the average number of deaths in the same month in 1911–1913, Italy [12].

Year	Month	Absolute Frequency of Excess (+ or −)
1918	June	−285
	July	−1201
	August	10,329
	September	77,999
	October	242,841
	November	118,142
	December	49,561
1919	April	25,461
	February	7069
	March	1055
	April	−3352
	May	−986

**Table 3:** The community mitigation strategies recommended by the ‘Popular Instructions’.

- Avoid the sick and the convalescents from the flu and their personal belongings
- Avoid public places and crowded means of transport
- Avoid disturbance and danger to neighbours (including raising dust, spitting on the floor or, better still, avoiding spitting altogether; coughing, sneezing or expectorating, if necessary, into one’s own handkerchief; getting into the habit of speaking without projecting droplets of saliva around oneself)
- Defend against unavoidable contact
- Intensify personal cleanliness (washing hands several times a day; rinsing the mouth with a mild antiseptic solution; gargling with alkaline detergent and carbolic acid and hydrogen peroxide-based disinfectants
- Keep the household clean
- Get into bed at the onset of symptoms and confine yourself to a room with little furniture and no carpets
- Disinfect objects used by patients

## Data Availability

No new data were created or analysed in this study. Data sharing does not apply to this article.
